# A Mitoxyperilysis‐Related Single‐Cell and Machine‐Learning Framework Defines an Immune‐Cold Melanoma Phenotype and a Robust Prognostic Signature

**DOI:** 10.1155/humu/9909803

**Published:** 2026-05-19

**Authors:** Yuze Zhou, Jiahong Fang, Lujing Fei, Huixian Li, Xiaolong Xu, Jiaheng Xie, Min Qi

**Affiliations:** ^1^ Department of Plastic Surgery, Xiangya Hospital, Central South University, Changsha, China, csu.edu.cn; ^2^ Department of Plastic Surgery, Shenzhen Hospital of Southern Medical University, Shenzhen, Guangdong, China

**Keywords:** machine learning, melanoma, mitoxyperilysis, single-cell RNA sequencing, tumor microenvironment

## Abstract

Mitoxyperilysis is a mitochondria‐dependent membrane lysis process driven by innate immune and metabolic cues, yet its clinical relevance in melanoma remains unclear. We analyzed single‐cell RNA‐seq data (GSE215120) to quantify a mitoxyperilysis‐related score (MRS), resolve cell‐type heterogeneity, and compare predicted cell–cell communication between MRS‐high and MRS‐low tumor states. MRS was robust across alternative scoring approaches and varied markedly across cell types and malignant subpopulations. Compared with MRS‐low tumors, the MRS‐high state exhibited increased predicted intercellular communication (740 vs. 448 interactions) and higher global interaction strength (18,989 vs. 10,652), suggesting a rewired tumor ecosystem. To translate these programs to bulk melanoma, we selected the top 150 genes most correlated with MRS and benchmarked 101 machine‐learning strategies in TCGA‐SKCM to derive prognostic models, followed by external validation in six independent GEO cohorts. A gradient boosting machine (GBM)–based signature showed the most consistent cross‐cohort performance and reliably stratified overall survival. High riskScore was associated with reduced immune and stromal signals, higher tumor purity, and an immune‐cold tumor microenvironment as estimated by multialgorithm deconvolution and ESTIMATE. As a representative model gene, GPR143 was upregulated in melanoma, was associated with worse survival, and its functional knockdown suppressed colony formation in melanoma cells. Collectively, this work establishes a novel integrative framework that—for the first time—connects single‐cell–resolved mitoxyperilysis‐associated transcriptional programs with large‐scale multicohort machine‐learning validation, thereby enabling both mechanistic interpretation of immunometabolic heterogeneity and clinically applicable risk stratification in melanoma.

## 1. Introduction

Cutaneous melanoma is one of the most aggressive skin cancers, characterized by high metastatic potential and substantial interpatient heterogeneity in clinical outcomes [1–3]. Despite major advances brought by immune checkpoint blockade and targeted therapies, a considerable proportion of patients experience primary resistance, relapse, or rapid progression [4, 5]. This variability highlights an ongoing need for prognostic frameworks that more faithfully capture both tumor‐intrinsic biology and the surrounding tumor microenvironment (TME), including immune and stromal components that critically shape disease trajectory and therapy response.

Mitochondria sit at the center of cellular stress responses, integrating metabolic cues with innate immune signaling [6–8]. Beyond their established roles in energy production and apoptosis, emerging evidence suggests that mitochondria can actively participate in distinct forms of regulated membrane damage [8]. In a recent study, Wang et al. reported that innate immune and metabolic perturbations can induce prolonged mitochondria–plasma membrane contacts (termed mitoxyperiosis) and trigger a mitochondria‐dependent membrane lysis phenotype (mitoxyperilysis) [9]. This work expands the landscape of lytic cell‐death mechanisms and suggests a conceptual link between immunometabolic stress, mitochondrial dynamics, and membrane integrity—processes that are highly relevant to melanoma biology, where metabolic adaptation and immune pressure coevolve during progression.

However, whether mitoxyperilysis‐related transcriptional programs are detectable in melanoma patient tissues, how such programs distribute across specific cellular compartments, and how they relate to microenvironmental states remain insufficiently explored. Single‐cell RNA sequencing provides a powerful approach to resolve pathway activity at cellular resolution, enabling the dissection of tumor cell states alongside immune and stromal populations [10–12]. In parallel, integrating mitoxyperilysis‐related signals into prognostic modeling may offer clinically useful risk stratification while providing mechanistic clues linked to TME phenotypes.

In this study, we therefore developed a mitoxyperilysis‐related score (MRS) to quantify mitoxyperilysis‐associated transcriptional features and investigated its cellular context using single‐cell data (GSE215120). We further sought to translate MRS‐associated gene patterns into a robust prognostic framework by training machine‐learning–based survival models in TCGA‐SKCM and validating them across multiple independent GEO cohorts. Finally, we aim to prioritize a key gene within the prognostic model for downstream validation, bridging predictive modeling with biological interpretation. Importantly, this work establishes a unified framework that integrates single‐cell–resolved mitoxyperilysis‐associated transcriptional programs with large‐scale multicohort machine‐learning validation—an approach not previously applied to mitoxyperilysis biology—thereby enabling both mechanistic interpretation and clinically relevant prognostic prediction.

## 2. Methods

### 2.1. Data Acquisition and Preprocessing

Single‐cell RNA‐seq data were obtained from GEO: GSE215120 [13]. Bulk transcriptomic profiles and clinical annotations for melanoma were downloaded for TCGA‐SKCM (training cohort) and multiple independent GEO cohorts (GSE19234, GSE22153, GSE53118, GSE54467, GSE59455, and GSE65904) for external validation. Additional GEO datasets (GSE46517 and GSE98394) were used to assess GPR143 expression differences between tumor and normal tissues and to evaluate associations with clinical stage. When necessary, probe‐level microarray data were mapped to gene symbols, and expression values were log2‐transformed and z‐score standardized within each cohort prior to downstream analyses.

### 2.2. Single‐Cell Analysis, Clustering, and Cell‐Type Annotation

Single‐cell analyses were performed using Seurat (R). Low‐quality cells were removed based on outlier filtering of detected genes/UMIs and mitochondrial transcript proportion: cells with < 200 detected genes, > 6000 detected genes (potential doublets), or mitochondrial transcript proportion > 15% were excluded. Thresholds were confirmed by examining the distribution of these metrics across all cells; cells falling outside the main peak regions were removed. Data were normalized and scaled, followed by identification of highly variable genes. Batch effects across samples were corrected using Seurat′s integration workflow (anchor‐based integration), and the integrated expression matrix was used for PCA, neighborhood graph construction, UMAP visualization, and graph‐based clustering.

Cell types were annotated using canonical marker genes, including:

• Epithelial/malignant: DCT, MLANA, MITF, PMEL;
• Endothelial: PECAM1, VWF;
• Fibroblasts: PDGFRA, LUM, COL1A1;
• Cycling: MKI67, STMN1;
• Myeloid: LYZ, CD68, CD74;
• B cells: MS4A1, CD79A, CD19;
• NK/T: GNLY, KLRD1, KLRC1, NCR1 (NK) and CD3D, CD3E, CD4, CD8A (T).

Cell‐type proportions were calculated per sample using annotated labels.

### 2.3. MRS Quantification in Single Cells

A mitoxyperilysis gene set (BAX/BAK1/BID, mTORC2) was curated based on prior literature and functional relevance to mitochondria‐dependent membrane lysis processes as described by Wang et al. [9]. The MRS was formally defined as a gene set–based enrichment score derived from a curated mitoxyperilysis‐related gene set obtained from Wang et al. [9], quantified using rank‐based and module‐scoring approaches (e.g., AddModuleScore, ssGSEA, and AUCell), representing the relative activity of mitoxyperilysis‐associated transcriptional programs at the single‐cell level.

### 2.4. Cell–Cell Communication Analysis

Intercellular communication was predicted using CellChat. Annotated single‐cell data were used to construct CellChat objects, identify overexpressed ligands/receptors, and compute communication probabilities and pathway‐level “information flow.” Communication networks were independently inferred for MRS_high and MRS_low groups, and differences in the number of interactions, overall interaction strength, and outgoing/incoming signaling roles were quantified. Pathway‐level comparisons were performed to identify signaling programs enriched in each MRS group.

### 2.5. Correlation Analysis and Candidate Gene Selection for Prognostic Modeling

To derive MRS‐associated candidates for prognostic modeling, genes were ranked by their correlation with MRS‐related metrics (MRS score) using Spearman correlation, followed by multiple‐testing correction (FDR). The top 150 correlated genes were retained as candidate features for subsequent machine‐learning model construction.

### 2.6. Machine‐Learning Construction of Prognostic Models

Using TCGA‐SKCM as the training cohort, we implemented an exhaustive survival‐modeling framework comprising 101 machine‐learning strategies, including 10 base algorithms (GBM, CoxBoost, random survival forest, elastic net, LASSO, Ridge, stepwise Cox, survival SVM, plsRcox, and SuperPC) and their hybrid combinations (e.g., Lasso + GBM and CoxBoost + GBM). For each method, hyperparameters were tuned within the training set using 10‐fold cross‐validation where applicable. Models were trained on the full TCGA cohort and then applied without refitting to each validation cohort. The workflow consisted of three steps: (1) feature selection using the top 150 MRS‐correlated genes; (2) model training in TCGA‐SKCM with cross‐validation across 101 strategies; and (3) external validation in six independent GEO cohorts without refitting. Performance was evaluated by concordance index (C‐index), Kaplan–Meier analysis, and time‐dependent ROC. For each method, a patient‐level riskScore was generated (model‐predicted risk or linear predictor), and patients were dichotomized into high‐ and low‐risk groups using the median riskScore within each cohort.

### 2.7. External Validation and Model Evaluation

The trained models were applied without refitting to six independent GEO cohorts (GSE19234, GSE22153, GSE53118, GSE54467, GSE59455, and GSE65904). Predictive performance was quantified using the C‐index. Kaplan–Meier survival analyses were performed using the log‐rank test, and hazard ratios were estimated by Cox proportional hazards regression. Time‐dependent ROC analyses were conducted to compute AUC values at 1, 3, and 5 years. To visualize separation of risk groups in an unsupervised manner, PCA was performed based on the expression of signature genes, and samples were plotted in PC space colored by risk group.

### 2.8. Benchmarking Against Published Prognostic Signatures

Previously published melanoma prognostic signatures were curated from PubMed (indexed by PMID) [14]. For each signature, risk scores were computed according to the original publication when coefficients/formulas were available; otherwise, the reported gene set was implemented using a standardized scoring strategy consistent across cohorts. C‐index values were calculated in TCGA‐SKCM and each GEO validation cohort to benchmark the performance of the final MRS‐GBM model against published methods.

### 2.9. Immune Infiltration and TME Analysis

Immune cell infiltration was estimated using multiple deconvolution methods, including TIMER, CIBERSORT, CIBERSORT‐ABS, quanTIseq, MCP‐counter, xCell, and EPIC (implemented via a unified pipeline). StromalScore, ImmuneScore, ESTIMATEScore, and tumor purity were computed using the ESTIMATE algorithm. Associations between riskScore and TME metrics were evaluated using Spearman correlation, with FDR adjustment for multiple testing. These analyses were exploratory in nature and based on the correlation between gene expression and drug sensitivity estimates, rather than direct experimental validation.

### 2.10. Identification and Validation of the Key Model Gene GPR143

To prioritize a key gene within the prognostic model, correlations between riskScore and the expression of model‐included genes were calculated, and GPR143 was selected based on its strong positive association with riskScore, its consistent performance across multiple datasets, and its inclusion as a stable component of the final prognostic model. Differential expression of GPR143 between normal and tumor tissues was assessed in GSE46517 and GSE98394 using appropriate statistical tests (Wilcoxon rank‐sum or t‐test, depending on distribution), and stage‐associated differences were evaluated using ANOVA. Protein‐level expression evidence was obtained from immunohistochemistry images in the Human Protein Atlas (HPA).

### 2.11. Functional Enrichment and Pathway Analysis

Genes associated with GPR143 (defined by correlation and FDR threshold) were subjected to GO and KEGG enrichment analyses using clusterProfiler. Enriched terms/pathways were considered significant at FDR‐adjusted *p* values below the predefined threshold.

### 2.12. Drug‐Response Association Analysis

Associations between GPR143 expression and drug response were evaluated using publicly available pharmacogenomic resources (e.g., GDSC/CTRP‐derived sensitivity metrics) and correlation‐based analyses. Drugs were categorized as potentially associated with sensitivity or resistance according to the direction of the association, with multiple‐testing correction applied.

### 2.13. Cell Culture, Gene Knockdown, and Colony Formation Assays

Human melanoma cell lines A375 and SK‐MEL‐28 were obtained from ATCC and cultured under recommended conditions. shRNA‐mediated knockdown of GPR143 (two independent shRNAs) and a nontargeting control were introduced using standard transfection/transduction procedures. For colony formation assays, equal numbers of cells were seeded into plates, cultured until visible colonies formed, fixed, stained with crystal violet, and quantified by colony counting. Group differences were assessed using two‐sided statistical tests.

### 2.14. Statistical Analysis

All statistical analyses were conducted in R. Unless stated otherwise, two‐sided tests were used, and multiple comparisons were corrected using the Benjamini–Hochberg method. A *p* (or *q*) value < 0.05 was considered statistically significant.

## 3. Results

MRS stratifies single‐cell states and is associated with enhanced intercellular communication.

After integrating four samples, UMAP visualization revealed 12 transcriptionally distinct Seurat clusters (Figure 1a). Cell‐type annotation resolved these clusters into six major compartments, including epithelial, NK/T, B, myeloid, endothelial, and fibroblasts populations (Figure 1b), with pronounced intersample differences in cellular composition (Figure 1c). Canonical marker expression supported the annotation: Epithelial cells expressed DCT/MLANA/MITF/PMEL; endothelial cells expressed PECAM1 and VWF; fibroblasts expressed PDGFRA/LUM/COL1A1; cycling cells were enriched for MKI67 and STMN1; myeloid cells expressed LYZ and CD68 (with CD74); B cells expressed MS4A1/CD79A/CD19; NK cells expressed GNLY/KLRD1/KLRC1/NCR1; and T cells expressed CD3D/CD3E together with CD4 and CD8A (Figure 1d). We next quantified the MRS at single‐cell resolution. MRS exhibited marked heterogeneity across the UMAP embedding (Figure 1e) and showed concordant patterns across multiple scoring strategies (AUCell, UCell, singscore, ssGSEA, AddModuleScore, and the applied scoring method), supporting the robustness of the MRS signal (Figure 1f). MRS distributions differed across cell types and displayed substantial within‐type variability, indicating intracompartment heterogeneity (Figure 1g). To assess whether MRS stratification relates to microenvironmental signaling, we predicted cell–cell communication in MRS_low versus MRS_high groups. MRS_high displayed a higher number of inferred interactions (740 vs. 448) and markedly increased global interaction strength (18,989 vs. 10,652) relative to MRS_low (Figure 1h). Sender–receiver profiling further indicated a coordinated increase in outgoing and incoming interaction strengths across multiple compartments in the MRS_high state, with fibroblasts showing prominent signaling output (Figure 1i). Pathway‐level analysis demonstrated broad remodeling of communication programs between MRS_low and MRS_high (Figure 1j), including immune/inflammatory and growth‐factor–related signals (e.g., TNF, TGFB, CSF, CCL, MIF, VEGF, and PDGF) and extracellular matrix/vascular adhesion‐associated pathways (e.g., FN1, COLLAGEN, LAMININ, ANGPT, VCAM, PECAM1, and SELE). Finally, genome‐wide correlation analysis ranked genes by their association with MRS (Figure 1k), providing a prioritized list of MRS‐linked candidates for downstream validation. Having established that MRS captures biologically meaningful variation in the single‐cell ecosystem—particularly its association with differential intercellular communication—we next sought to determine whether MRS‐associated transcriptional programs could be translated to bulk tumors for prognostic stratification. This translational step required selecting genes whose expression consistently reflected MRS activity across cellular contexts, as these might capture the multifaceted impact of mitoxyperilysis‐related programs on patient outcomes.

Figure 1Single‐cell atlas of melanoma and mitoxyperilysis‐related score (MRS) reveals heterogeneous activity and differential cell–cell communication. (a) UMAP embedding of integrated single‐cell transcriptomes from GSE215120 showing 12 Seurat clusters. (b) UMAP annotated by major cell types (epithelial, NK/T, B, myeloid, endothelial, and fibroblasts). (c) Cell‐type composition across samples (GSM6622299–GSM6622302). (d) Dot plot of canonical marker genes used for cell‐type annotation. (e) UMAP feature plot showing the distribution of MRS at single‐cell resolution. (f) Comparison of MRS estimated by multiple scoring approaches (AUCell, UCell, singscore, ssGSEA, AddModuleScore, and the applied scoring method). (g) Violin plots showing MRS distributions across cell types. (h) CellChat summary comparing inferred interaction number and global interaction strength between MRS_low and MRS_high (interaction number: 448 vs. 740; global strength: 10,652 vs. 18,989). (i) Outgoing and incoming communication strength across cell types in MRS_low and MRS_high. (j) Differential pathway‐level information flow between MRS_low and MRS_high inferred by CellChat. (k) Genome‐wide ranking of genes by correlation with MRS‐based scoring for downstream candidate selection.

**Figure:**
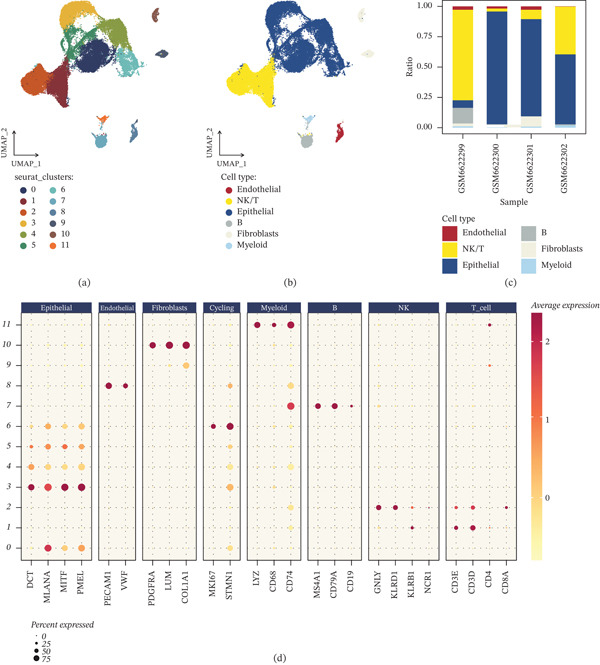

**Figure:**
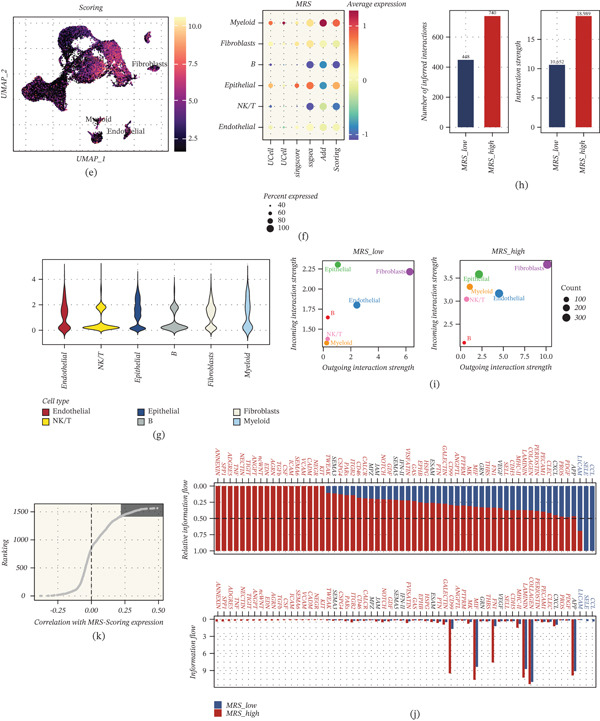

### 3.1. Machine‐Learning Construction and External Validation of an MRS‐Correlated Prognostic Model

**Figure 2:**
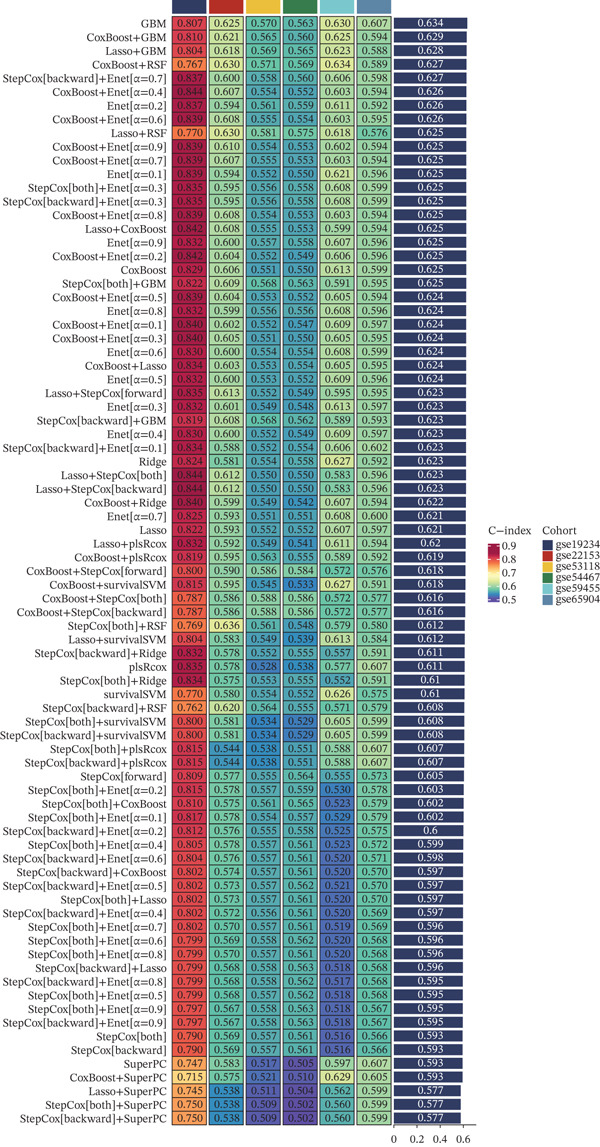
Comprehensive machine‐learning screening identifies an optimal MRS‐derived prognostic model. Heatmap showing the concordance index (C‐index) of 101 survival modeling strategies trained in TCGA‐SKCM using the top 150 MRS‐correlated genes and evaluated across independent GEO cohorts (GSE19234, GSE22153, GSE53118, GSE54467, GSE59455, and GSE65904). Rows indicate algorithms/algorithm combinations and columns indicate cohorts; the right bar summarizes mean C‐index across validation cohorts. The best‐performing method (GBM) is highlighted.

Based on the premise that genes strongly correlated with MRS at single‐cell resolution may serve as stable proxies for mitoxyperilysis‐related activity in bulk tissue, we proceeded to construct prognostic models using the top 150 MRS‐correlated genes. Using an exhaustive machine‐learning framework, a total of 101 survival‐modeling strategies (including GBM, CoxBoost, random survival forest, elastic net, LASSO/Ridge, stepwise Cox, survival SVM, plsRcox, and multiple hybrid combinations) were trained in the TCGA cohort and then externally validated across six independent GEO cohorts (GSE19234, GSE22153, GSE53118, GSE54467, GSE59455, and GSE65904). Model discrimination was quantified by the C‐index. The heatmap summarizes the C‐index achieved by each method in each validation cohort, with the right‐side bar indicating the mean C‐index across cohorts (Figure 2). Among all candidate methods, the GBM‐based model showed the best overall generalization ability, yielding the highest mean C‐index (0.634), followed by CoxBoost + GBM (0.629) and Lasso + GBM (0.628). In contrast, SuperPC‐based approaches showed comparatively lower mean performance (~0.577), suggesting reduced cross‐cohort robustness in this setting (Figure 2).

### 3.2. Benchmarking Against Published Prognostic Signatures Demonstrates Superior Performance of the MRS Model

**Figure 3:**
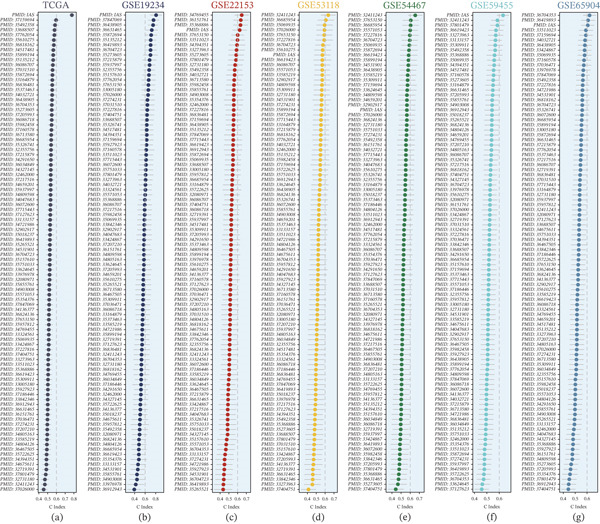
Benchmarking the MRS‐GBM model against published prognostic signatures curated from PubMed. Forest plots compare C‐index (with confidence intervals) for the final MRS‐GBM model versus previously published prognostic signatures (annotated by PMID) in (a) TCGA‐SKCM and (b–g) GEO validation cohorts (GSE19234, GSE22153, GSE53118, GSE54467, GSE59455, and GSE65904). Higher C‐index indicates better discrimination.

To place our final GBM‐derived prognostic model based on MRS–correlated genes in context, we systematically benchmarked it against previously published prognostic signatures curated from PubMed (indexed by PMID). Using a unified evaluation pipeline, we calculated the C‐index (with corresponding confidence intervals) for each signature in the TCGA cohort (Figure 3a) and across six independent GEO validation cohorts (GSE19234, GSE22153, GSE53118, GSE54467, GSE59455, and GSE65904; Figure 3b–g). In the TCGA cohort, the MRS model ranked among the best‐performing signatures in terms of concordance (Figure 3a). Importantly, this competitive advantage was maintained during external validation, where the MRS model consistently achieved high C‐index values and remained near the top across all GEO cohorts (Figure 3b–g), supporting the robustness and generalizability of our MRS‐based prognostic prediction.

### 3.3. The MRS‐GBM Prognostic Signature Robustly Stratifies Survival Risk and Shows Stable Predictive Accuracy Across Cohorts

The final GBM‐based prognostic signature derived from MRS‐correlated genes effectively separated patients into high‐ and low‐risk groups with significantly different survival outcomes in the TCGA training cohort and all six GEO validation cohorts (Figure 4a). Specifically, Kaplan–Meier analyses showed markedly worse survival in the high‐risk group, with significant log‐rank *p* values in TCGA (*p* < 0.0001) and the external datasets (GSE19234, *p* < 0.0001; GSE22153, *p* = 0.00093; GSE53118, *p* = 0.026; GSE54467, *p* = 0.012; GSE59455, *p* = 1*e* − 04; GSE65904, *p* = 0.00049) (Figure 4a). Time‐dependent ROC curves further supported the prognostic performance of the model (Figure 4b), achieving AUCs of 0.81/0.80/0.82 at 1/3/5 years in TCGA, and maintaining predictive capability across GEO cohorts (GSE19234: 0.69/0.89/0.85; GSE22153: 0.75/0.68/0.61; GSE53118: 0.77/0.54/0.59; GSE54467: 0.71/0.53/0.59; GSE59455: 0.77/0.73/0.62; and GSE65904: 0.60/0.68/0.65) (Figure 4b). In addition, PCA based on the signature‐related expression patterns demonstrated an overall separation trend between high‐ and low‐risk patients across cohorts, indicating that the risk groups occupy distinct transcriptional spaces and providing an unsupervised validation of the model′s discriminative capacity (Figure 4c).

Figure 4The MRS‐GBM risk score stratifies survival and demonstrates predictive accuracy across training and validation cohorts. (a) Kaplan–Meier survival curves comparing high‐ and low‐risk groups defined by the MRS‐GBM riskScore in TCGA‐SKCM and each GEO cohort; *p* values were calculated by the log‐rank test. (b) Time‐dependent ROC curves showing AUC at 1, 3, and 5 years for TCGA‐SKCM and each GEO cohort. (c) Principal component analysis (PCA) based on the prognostic signature genes showing the distribution of high‐ and low‐risk patients in each cohort.

**Figure:**
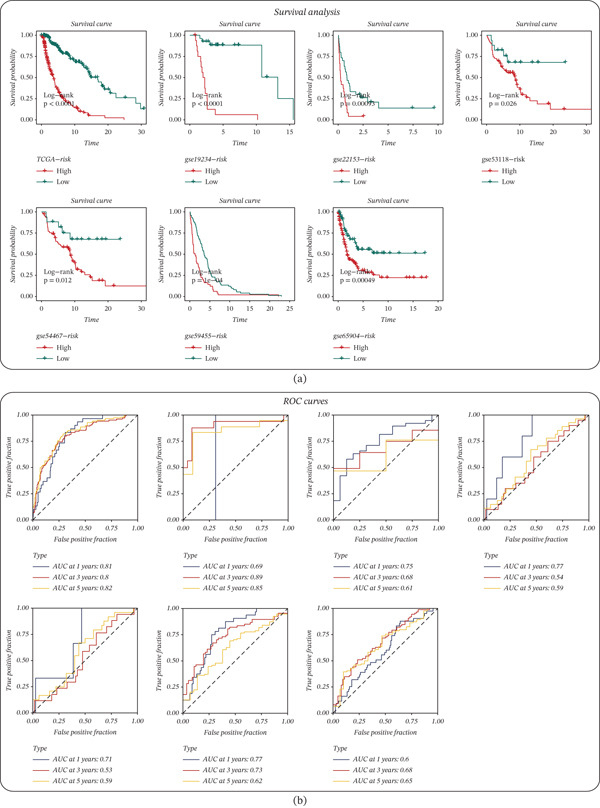

**Figure:**
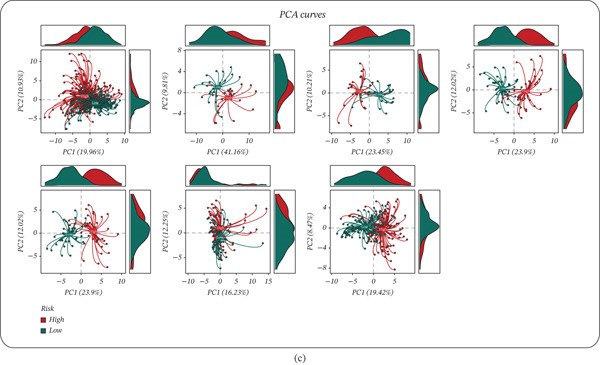

### 3.4. The MRS‐Based Risk Signature Is Associated With an Immunologically “Cold” TME and Increased Tumor Purity

To characterize TME differences between risk groups defined by the MRS‐GBM prognostic signature, we quantified immune infiltration using multiple deconvolution algorithms. Across TIMER, CIBERSORT, CIBERSORT‐ABS, quanTIseq, MCP‐counter, xCell, and EPIC, the high‐risk group showed broadly reduced immune cell infiltration compared with the low‐risk group, whereas the low‐risk group displayed a higher abundance of multiple immune populations (Figure S1A). Consistently, correlation analyses demonstrated that riskScore was negatively associated with stromalScore (*r* = −0.42, *q* = 0), immuneScore (*r* = −0.55, *q* = 0), and ESTIMATEScore (*r* = −0.54, *q* = 0), but positively correlated with tumor purity (*r* = 0.55, *q* = 0) (Figure S1B). Collectively, these results indicate that elevated MRS‐derived risk is linked to a tumor‐cell–dominant microenvironment with reduced immune and stromal components.

### 3.5. Identification of GPR143 as a Key Gene Within the MRS‐Based Prognostic Model

**Figure 5:**
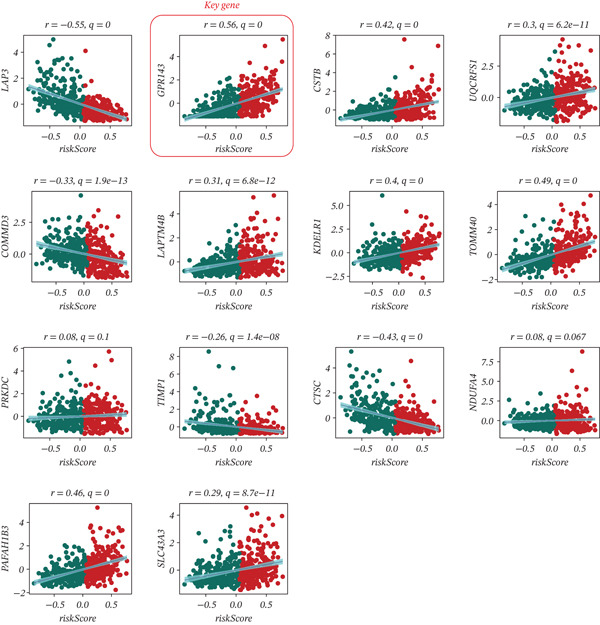
Identification of GPR143 as a key component gene within the prognostic model. Scatter plots showing correlations between expression of model‐included genes and the MRS‐GBM riskScore, highlighting genes with positive or negative associations. GPR143 is emphasized as a representative key gene due to its strong positive correlation with riskScore (*r* and *q* shown in the panel).

To identify a representative key gene from the MRS‐based prognostic signature, we focused on genes included in the final prognostic model and evaluated their relationships with the model‐derived riskScore. Among the model components, multiple genes showed significant correlations with riskScore, including negatively correlated genes (e.g., LAP3, COMMD3, TIMP1, and CTSC) and positively correlated genes (e.g., CSTB, UQCRFS1, LAPTM4B, KDELR1, TOMM40, PAFAH1B3, and SLC43A3) (Figure 5). Notably, GPR143, which is one of the genes comprising the prognostic model, exhibited one of the strongest positive associations with riskScore (*r* = 0.56, *q* = 0) (Figure 5). Therefore, GPR143 was selected as the key model gene for subsequent mechanistic and validation analyses.

### 3.6. Expression, Prognostic Value, and Functional Relevance of the Model Gene GPR143 in Melanoma

To validate GPR143 as a key component gene within the MRS‐based prognostic model, we first examined its expression patterns in independent datasets. GPR143 was significantly upregulated in tumor tissues compared with normal controls in both GSE46517 (Wilcoxon *p* = 0.012; Figure 6a) and GSE98394 (t‐test *p* = 0.0067; Figure 6b). Moreover, GPR143 expression increased with clinical stage in GSE98394 (ANOVA *p* = 0.008; Figure 6c). At the protein level, immunohistochemistry images from the HPA further supported stronger GPR143 staining in melanoma tissues than in normal skin (Figure 6d). To explore potential biological functions associated with GPR143, we performed enrichment analyses based on GPR143‐associated gene sets. GO terms were prominently enriched in pigmentation‐related processes (e.g., melanogenesis/pigmentation, melanosome, and pigment granule components) as well as membrane‐ and adhesion‐related features (Figure 6e). KEGG analysis further indicated enrichment of pathways linked to cancer and microenvironmental signaling, including apoptosis, focal adhesion/ECM–receptor interaction, cytokine‐related signaling, and melanoma‐related pathways (Figure 6f). Clinically, univariate Cox regression analyses across multiple cohorts and endpoints indicated that higher GPR143 expression was generally associated with increased risk (Figure 6g). Kaplan–Meier analyses consistently showed worse survival in the GPR143‐high group, including overall survival in TCGA‐SKCM (log‐rank *p* = 0.0036; Figure 6h) and disease‐specific survival (log‐rank *p* = 0.013; Figure 6i), with external validation in GSE19234 (log‐rank *p* = 0.023; Figure 6j) and GSE98394 (log‐rank *p* = 0.00044; Figure 6k). In addition, drug‐response association analysis suggested that GPR143 expression may stratify sensitivity versus resistance patterns across multiple agents, highlighting its potential therapeutic relevance (Figure 6l).

Figure 6Validation of GPR143 expression, prognostic significance, and functional relevance in melanoma. (a–b) GPR143 expression in tumor versus normal samples in GEO datasets (GSE46517 and GSE98394). (c) Association of GPR143 expression with clinical stage in GSE98394. (d) Representative immunohistochemistry images of GPR143 protein expression in normal skin and melanoma tissues (Human Protein Atlas). (e–f) GO and KEGG enrichment analyses of GPR143‐associated genes. (g) Univariate Cox regression summarizing hazard ratios of GPR143 across cohorts/endpoints. (h–k) Kaplan–Meier survival analyses comparing GPR143‐high versus GPR143‐low groups in TCGA‐SKCM and GEO cohorts (log‐rank test). (l) Association of GPR143 expression with drug response metrics (sensitivity/resistance patterns) in pharmacogenomic analysis. (m–n) Colony formation assays following shRNA‐mediated GPR143 knockdown in A375 and SK‐MEL‐28 cells, with representative images and quantification.

**Figure:**
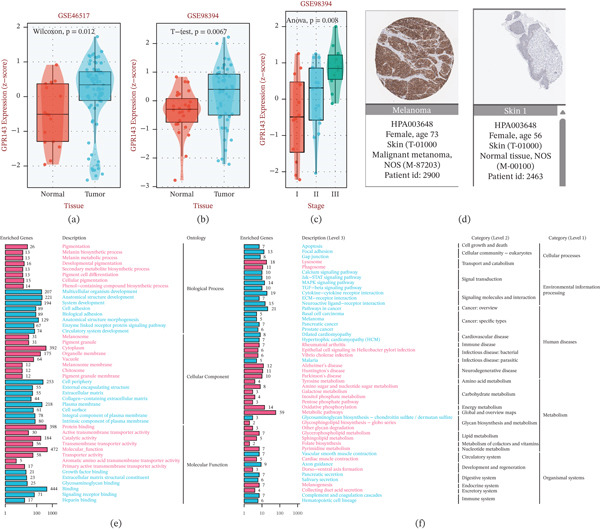

**Figure:**
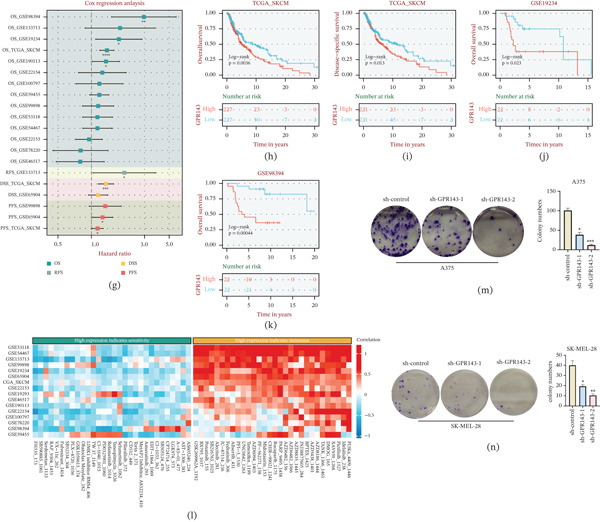

Finally, functional experiments supported an oncogenic role for GPR143: shRNA‐mediated knockdown of GPR143 reduced clonogenic growth in both A375 and SK‐MEL‐28 melanoma cells, with a marked decrease in colony numbers compared with sh‐control (Figure 6m–n). Collectively, these data validate GPR143 as a model‐included key gene that is upregulated in melanoma, predicts poor prognosis, and promotes tumor cell growth.

## 4. Discussion

Our study connects the emerging concept of mitochondria‐dependent membrane lysis—mitoxyperilysis—to melanoma by building a MRS and integrating single‐cell context with multicohort prognostic modeling. Mechanistically, mitoxyperilysis was recently described as a lytic outcome driven by sustained mitochondria–plasma membrane contacts (mitoxyperiosis) under innate immune and metabolic cues, broadening the landscape of regulated membrane damage and highlighting mitochondria as active executors of stress‐linked cell death [9]. Melanoma evolves under strong immunologic pressure and metabolic constraint, making it a compelling setting in which such an immunometabolic, mitochondria‐centered program might leave a detectable transcriptional footprint and relate to clinical heterogeneity [15–17].

At single‐cell resolution, MRS exhibited pronounced heterogeneity across the tumor ecosystem, differing between major compartments and varying substantially within each compartment. The concordance across multiple scoring approaches argues that the observed pattern is not driven by a single computational choice but reflects a stable signal in the data. This behavior is biologically consistent with stress‐response programs that depend on local nutrient and oxygen availability, inflammatory cytokines, and microenvironmental interactions. Importantly, when stratifying by MRS, the computationally inferred intercellular communication landscape shifted markedly. The MRS_high condition showed both more predicted interactions and higher global interaction strength (740 vs. 448; 18,989 vs. 10,652), accompanied by broad pathway‐level remodeling that included immune/inflammatory signals, growth‐factor programs, and ECM/adhesion‐associated pathways. While ligand–receptor inference does not prove physical signaling events, the coherence of the changes suggests that MRS_high tumors may represent a more “networked” state in which tumor and stromal/immune compartments engage in intensified crosstalk, potentially reflecting adaptation to stress or microenvironmental restructuring that supports tumor persistence.

Translating MRS‐associated transcriptional variation to bulk tumors, we constructed prognostic models from the top 150 MRS‐correlated genes using a comprehensive machine‐learning framework. The final GBM‐based model generalized well across multiple independent GEO cohorts and remained competitive when benchmarked against previously published signatures. The robustness across platforms and cohorts is notable, because many reported signatures degrade substantially outside the discovery dataset due to batch effects, cohort composition, and differences in clinical annotation. The strong mean performance of the GBM approach also implies that nonlinear interactions among MRS‐linked features may carry prognostic information beyond what strictly linear models can capture, which is plausible in melanoma given the intertwined nature of mitochondrial metabolism, stress signaling, and microenvironmental regulation.

A consistent biological interpretation emerged from the TME analyses: high riskScore aligned with features suggestive of an immunologically “cold‐like,” tumor‐cell–dominant phenotype [18]. RiskScore negatively correlated with stromalScore, immuneScore, and ESTIMATEScore and positively correlated with tumor purity, and multialgorithm deconvolution broadly supported reduced immune infiltration in the high‐risk group. This coupling has several plausible explanations. MRS‐high, high‐risk tumors might engage stress‐adaptive programs that favor immune evasion or immune exclusion, for example by altering cytokine networks, antigen presentation, or ECM remodeling [19–21]. Conversely, immune‐ and stroma‐poor environments may impose metabolic constraints that amplify mitochondrial stress and related transcriptional programs, producing the observed association without a direct causal arrow from MRS to immune depletion [22]. A third possibility is a feedback loop in which stromal architecture and fibroblast‐associated ECM restrict immune trafficking while simultaneously shaping tumor metabolic states, and tumor cells in turn reinforce exclusionary microenvironmental cues through paracrine signaling. Distinguishing these alternatives will require spatially resolved measurements and perturbation experiments that can separate tumor‐intrinsic effects from composition‐driven confounding.

To bridge prediction to mechanism, we prioritized GPR143, a component gene within the prognostic model, because its expression was strongly positively associated with riskScore and it demonstrated stable behavior across datasets within the prognostic model. GPR143 is particularly compelling in melanoma given its connection to melanocytic lineage biology and pigment organelles [23]. Across independent datasets, GPR143 was upregulated in tumor compared with normal tissue, increased with clinical stage, and displayed protein‐level support by immunohistochemistry, whereas survival analyses consistently associated high GPR143 with poor outcomes. Functional assays further showed that shRNA‐mediated knockdown of GPR143 reduced clonogenic growth in A375 and SK‐MEL‐28 cells, supporting a protumor role. Enrichment analyses linked GPR143‐associated programs to pigmentation/melanosome processes as well as adhesion/ECM and cancer‐related signaling pathways, providing a reasonable biological scaffold connecting GPR143 to invasive behavior and microenvironmental interactions. Nevertheless, the mechanistic relationship between GPR143 and mitoxyperilysis remains to be established; it is possible that GPR143 acts upstream by shaping organelle biology and stress adaptation, downstream as a marker of a broader melanoma state, or in parallel through microenvironmental remodeling. Given the mitochondria‐centered nature of mitoxyperilysis, future work should test whether GPR143 perturbation alters mitochondrial dynamics, oxidative metabolism, innate immune signaling nodes, or susceptibility to mitochondria‐dependent membrane damage.

Several limitations should be considered when interpreting these findings. First, all analyses are retrospective in nature; although we employed multicohort validation, prospective evaluation in clinically annotated cohorts with treatment information will be essential to establish clinical utility. Second, the single‐cell conclusions rely on a single dataset (GSE215120); validating the cellular distribution of MRS and inferred signaling shifts across additional scRNA‐seq and spatial transcriptomics datasets would strengthen generality. Third, cell–cell communication analyses are inference‐based and do not prove physical signaling events; orthogonal validation using spatial transcriptomics, multiplex imaging, or functional ligand–receptor perturbation is needed to confirm the proposed interaction rewiring. Fourth, MRS is transcriptome‐derived, whereas mitoxyperilysis is defined by dynamic organelle–membrane contacts and membrane lysis; transcriptional proxies may not fully capture posttranscriptional regulation or the temporal nature of the process. Finally, while we benchmarked against published signatures, differences in preprocessing and cohort composition can influence comparative performance; standardized prospective head‐to‐head testing would provide the strongest evidence.

Overall, our results suggest that mitoxyperilysis‐related transcriptional programs are measurable in melanoma, associate with distinct cellular states and communication patterns, and can be leveraged to build a robust prognostic model linked to an immune‐depleted TME. The identification and functional validation of GPR143 provide an entry point for mechanistic exploration and potential therapeutic stratification. More broadly, this study supports the premise that newly defined immunometabolic death pathways can be translated into clinically relevant tumor signatures, motivating future work to connect mitochondrial stress biology with microenvironmental remodeling and treatment response in melanoma.

## Author Contributions

Yuze Zhou, Jiahong Fang, Lujing Fei, Huixian Li, Xiaolong Xu, Jiaheng Xie, and Min Qi made a significant contribution to the work reported, whether that is in the conception, study design, execution, acquisition of data, analysis, and interpretation, or in all these areas; took part in drafting, revising, or critically reviewing the article. Yuze Zhou, Jiahong Fang, and Lujing Fei contributed equally to this work.

## Funding

This study was supported by National Natural Science Foundation of China (10.13039/501100001809; 82073019).

## Disclosure

All authors gave final approval of the version to be published, agreed on the journal to which the article has been submitted, and agreed to be accountable for all aspects of the work.

## Ethics Statement

This study was performed in accordance with the Declaration of Helsinki. Human cell lines included in this study were approved as part of this study protocol. This study was approved by the Ethics Committee of Xiangya Hospital of Central South University (2024030015).

## Conflicts of Interest

The authors declare no conflicts of interest.

## Supporting information


**Supporting Information** Additional supporting information can be found online in the Supporting Information section. Figure S1: Association of the MRS‐GBM risk score with immune infiltration and tumor microenvironment features. (A) Heatmap summarizing immune cell infiltration estimates for low‐ and high‐risk groups computed by multiple deconvolution algorithms (TIMER, CIBERSORT, CIBERSORT‐ABS, quanTIseq, MCP‐counter, xCell, and EPIC). (B) Correlation analysis between riskScore and ESTIMATE‐derived metrics (stromalScore, immuneScore, ESTIMATEScore, and tumor purity). Correlation coefficients (*r*) and adjusted *p* values (*q*) are shown.

## Data Availability

The datasets analyzed in this study were obtained from public repositories, including The Cancer Genome Atlas (TCGA; https://portal.gdc.cancer.gov/) and the Gene Expression Omnibus (GEO; https://www.ncbi.nlm.nih.gov/geo/) under Accession Numbers GSE215120, GSE19234, GSE22153, GSE53118, GSE54467, GSE59455, and GSE65904.
