# Supplementary figures and images for: A Mitoxyperilysis‐Related Single‐Cell and Machine‐Learning Framework Defines an Immune‐Cold Melanoma Phenotype and a Robust Prognostic Signature
Source: Hum Mutat. 2026 May 19;2026:9909803. doi: 10.1155/humu/9909803 (PMC13189447; doi:10.1155/humu/9909803)

A

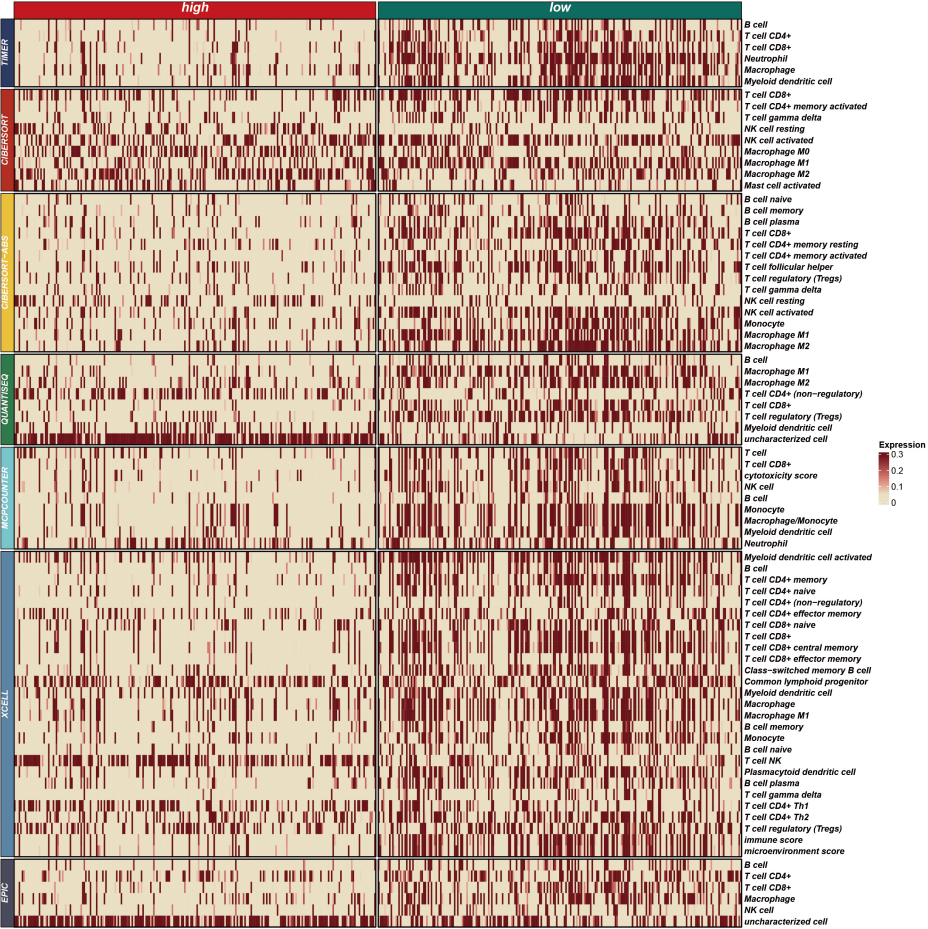

B

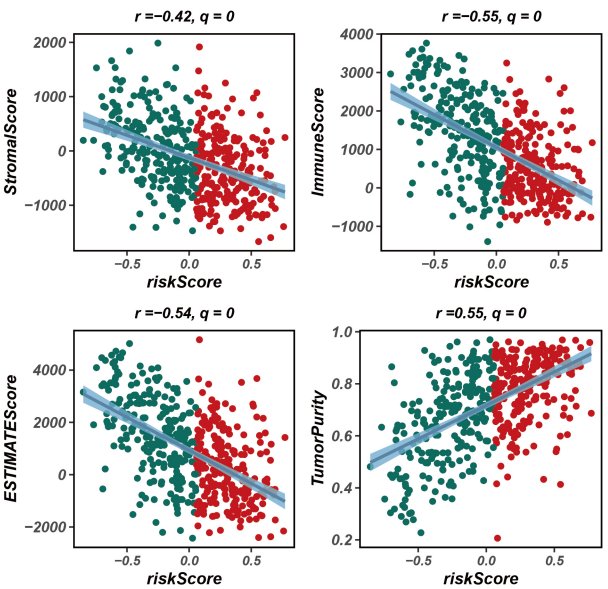

Supplement: Supplementary file 1 — Supporting Information Additional supporting information can be found online in the Supporting Information section. Figure S1: Association of the MRS‐GBM risk score with immune infiltration and tumor microenvironment features. (A) Heatmap summarizing immune cell infiltration estimates for low‐ and high‐risk groups computed by multiple deconvolution algorithms (TIMER, CIBERSORT, CIBERSORT‐ABS, quanTIseq, MCP‐counter, xCell, and EPIC). (B) Correlation analysis between riskScore and ESTIMATE‐derived metrics (stromalScore, immuneScore, ESTIMATEScore, and tumor purity). Correlation coefficients (r) and adjusted p values (q) are shown. [file HUMU-2026-9909803-s001.pdf]
